# Supplementary material for: Quasi-continuous transition from a Fermi liquid to a spin liquid in κ-(ET)2Cu2(CN)3
Source: Nat Commun. 2018 Jan 22;9:307. doi: 10.1038/s41467-017-02679-7 (PMC5778024; doi:10.1038/s41467-017-02679-7)
Supplement: Supplementary file 1 — Supplementary Information [file 41467_2017_2679_MOESM1_ESM.pdf]

Supplementary Note 1

Determination of internal pressure below the helium solidification temperature

The *P*-*T* phase diagram of helium is displayed in Supplementary Fig. 1. Using helium as a pressure medium, we varied pressure continuously in the *P*-*T* region above the solidification line. Below that line, the pressure was inevitably fixed, and only temperature variations were available in the experiments. As described in the Methods, the internal pressure was reduced by approximately 10–30 MP on the solidification of helium in the present pressure apparatus. There was a small difference between the internal pressure in the solid helium and the indication provided by the pressure gauge, which measured the pressure of the helium gas linked to solid helium through a tube (note that they do not differ when helium is in a liquid or gas state). Nevertheless, we could precisely estimate the internal pressure by monitoring the resistance of the sample by the following method. Supplementary Fig. 2 shows examples of the resistance behaviours (of sample #1) on cooling across the helium solidification temperature. The helium began to solidify at a temperature indicated by the arrow labelled R_{solid}, and the solidification was completed at the temperature indicated by R_{solid}'. Between the two temperatures, the internal pressure was gradually reduced along the solidification curve, and the resistance of the sample increased; at lower temperatures, the internal pressure was fixed. We determined the internal pressures at the temperatures marked by R_{solid}' and below by the following procedure. First, we measured the resistance on the solidification curve, $R_{solid}(P)$, by reading the pressure and resistance values just before solidification, P_{solid} and R_{solid}, which should be equal to the internal pressure and the resistance at the indicated pressure, respectively, on the solidification line (Supplementary Fig. 2). Second, we fitted the P_{solid} vs. R_{solid} data using a fifth-order polynomial, which gave the form of $R_{solid}(P)$ on the solidification curve. Finally, by using the fitting curve of $R_{solid}(P)$, we determined the internal pressure, P_{solid}', which yielded the resistance just after the completion of the helium solidification, R'_{solid}.

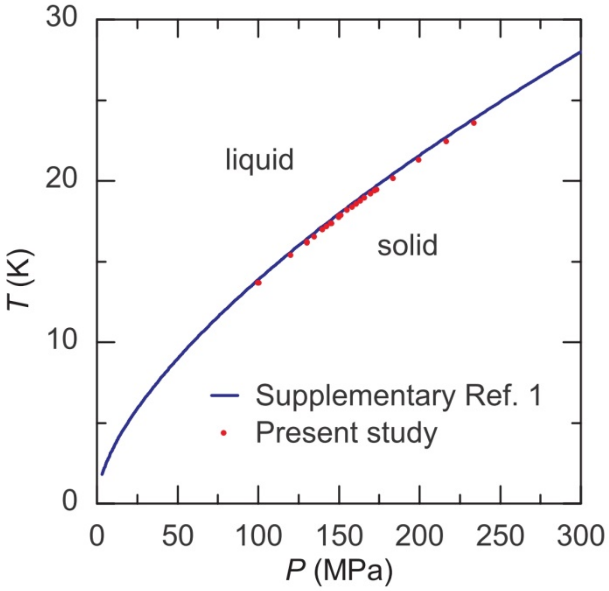

Supplementary Figure 1 | Phase diagram of helium. The red points indicate the pressure and temperature values measured just before solidification. The blue line was reproduced from Supplementary Ref. 1.

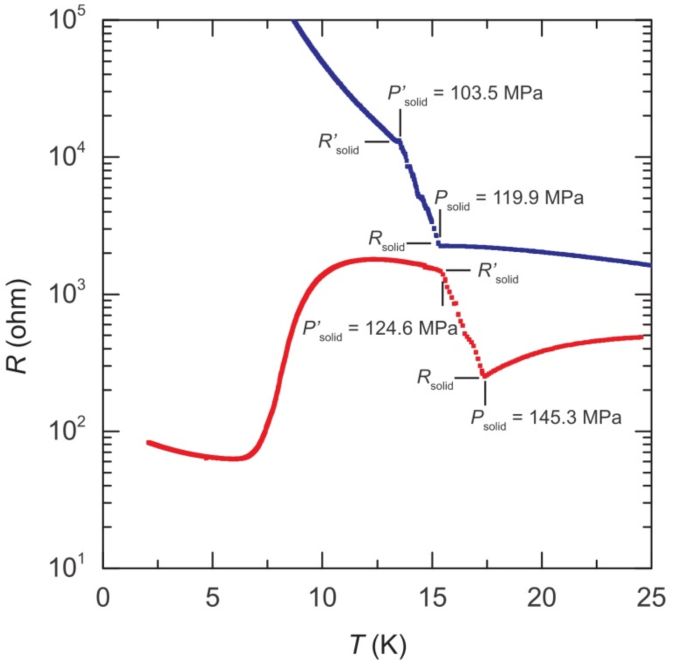

Supplementary Figure 2 | Spurious change in resistance caused by a pressure drop around the melting point of the helium pressure medium. The red and blue curves are examples of the temperature dependencies of resistance across the solidification temperature of helium.

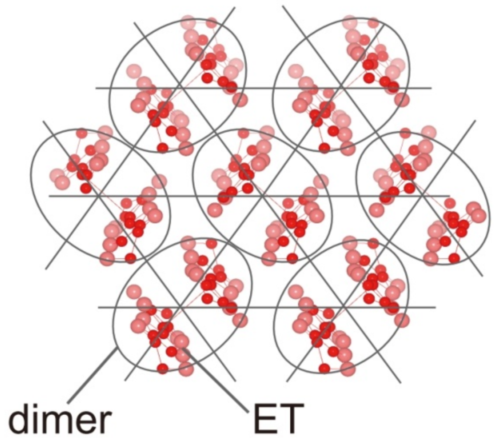

Supplementary Figure 3 | Structure of a conducting layer in κ-$(ET)_2Cu_2(CN)_3$. The conducting layers comprised of $(ET)_2$ dimers are stacked alternately with the non-magnetic insulating $Cu_2(CN)_3$ layers. A $(ET)_2$ dimer plays the role of one lattice point of an anisotropic triangular lattice and accommodates one hole. Due to the strong on-site Coulomb repulsion, this material is a Mott insulator at ambient pressure.

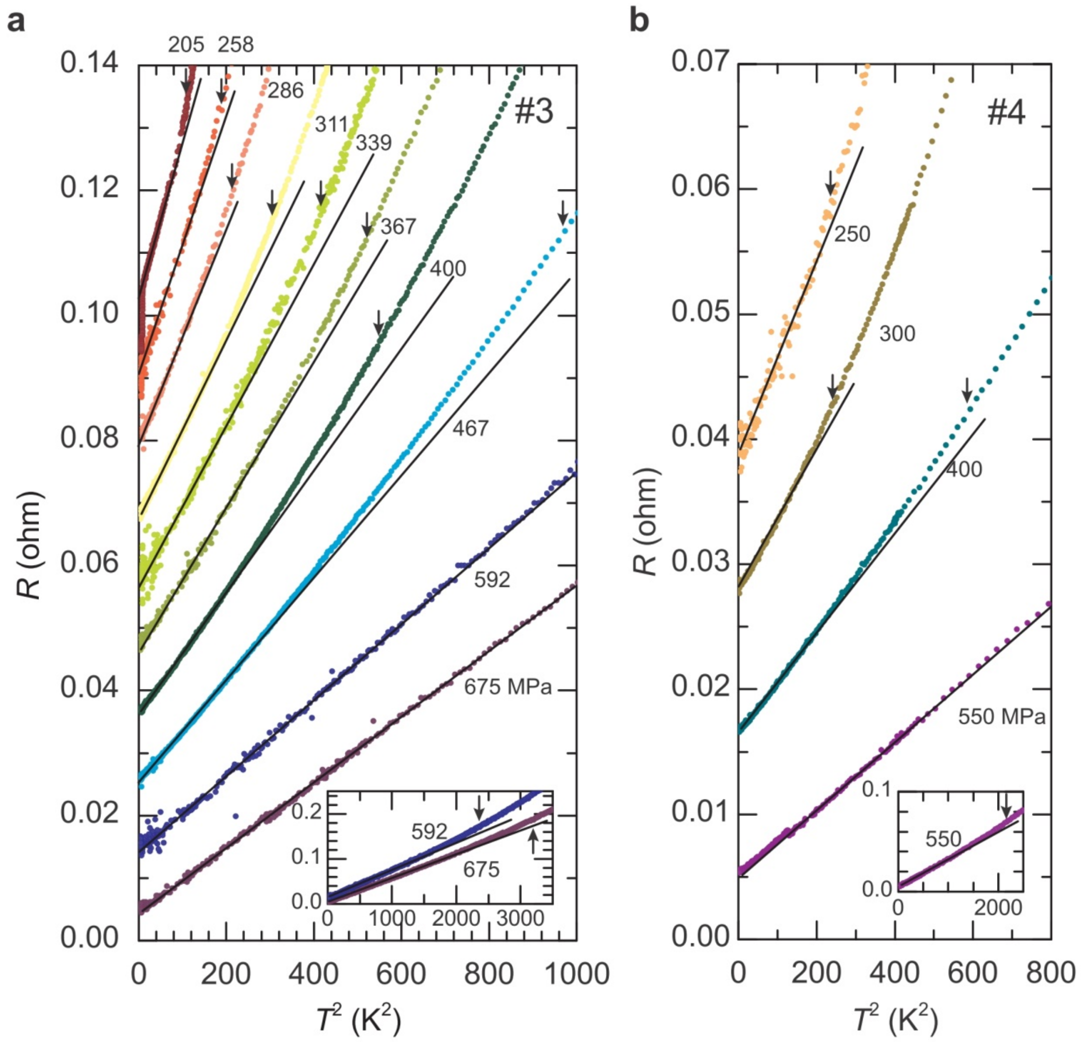

Supplementary Figure 4 | Resistance vs. T^2 for sample #3 (a) and sample #4 (b). The resistance is plotted with a vertical offset of the 0.01 ohm interval for clarity. The solid lines are the fits of equation $R(T) = R_0 + AT^2$ to the low-temperature parts of the data. The arrows indicate T^{*2} at each pressure.

Supplementary Note 2

Wilson ratio of the metallic κ-(ET)$_2$*X* situated on the Mott boundary

We used the value of the Wilson ratio R_W of the κ-(ET)$_2$*X* metallic phase neighbouring the Mott insulating phase in the analysis to deduce the spin entropy in κ-(ET)$_2$Cu$_2$(CN)$_3$. R_W is given by $R_W = (\pi^2 k_B^2/3\mu_B^2) \times (\chi/\gamma)$, where k_B is the Boltzmann constant, μ_B is the Bohr magneton, χ is spin susceptibility, and γ is the electronic specific heat coefficient. The experimental data for χ and γ are available for two compounds with X = Cu(NCS)$_2$ and Cu[N(CN)$_2$]Br. For X = Cu(NCS)$_2$, a γ of ~ 24 mJ K^{-2} mol^{-1} (Supplementary Ref. 2) and a χ_{metal} of ~ 3.2 × 10^{-4} emu mol^{-1} (Supplementary Ref. 3) yield an R_W of ~ 0.97. For X = Cu[N(CN)$_2$]Br], a γ of ~ 22 mJ K^{-2} mol^{-1} (Supplementary Ref. 4) and a χ_{metal} of ~ 3.0 × 10^{-4} emu mol^{-1} (Supplementary Ref. 5) yield an R_W of ~ 0.99. Thus, R_W of the κ-(ET)$_2$*X* metallic phase is assumed to be approximately unity.

Supplementary Note 3

Volume change Δ*V* on the Mott transition in κ-(ET)$_2$Cu[N(CN)$_2$]Cl

Supplementary Fig. 5(a) shows the temperature-pressure phase diagram of κ-(ET)$_2$Cu[N(CN)$_2$]Cl reproduced from Supplementary Ref. 6. Supplementary Fig. 5(b) shows a fitting curve of the first-order Mott transition points in Fig. 5(a). In the main text, we conducted a Clausius-Clapeyron analysis for κ-(ET)$_2$Cu[N(CN)$_2$]Cl using the slope of the transition curve in Supplementary Fig. 5(b).

As described in the main text, the Clausius-Clapeyron analysis for κ-(ET)$_2$Cu[N(CN)$_2$]Cl provided an evaluation of the molar volume change at the first-order Mott transition, Δ*V*, which yielded 4−6 × 10^{-7} m^3 mol^{-1}. On the other hand, Δ*V* in κ-(ET)$_2$Cu[N(CN)$_2$]Cl can be independently evaluated using the expansivity data[7]. The volume change on the Mott transition is approximately expressed by $\Delta V/V \sim \Delta l_a/l_a + \Delta l_b/l_b$, where $l_{i = a, b}$ and $\Delta l_{i = a, b}$ are the molar length of a sample and its change on the Mott transition measured along the in-plane *a* axis and the out-of-plane *b* axis, respectively. According to Supplementary Ref. 7, $\Delta l_c/l_c$ is negligible compared with $\Delta l_a/l_a$ and $\Delta l_b/l_b$; namely, $\Delta l_c/l_c << \Delta l_a/l_a, \Delta l_b/l_b$. Referring to Fig. 2 in Supplementary Ref. 7, $\Delta l_a/l_a \sim 3.2 \times 10^{-4}$ and $\Delta l_b/l_b \sim 2.3 \times 10^{-4}$ on the Mott-transition at 22 MPa and 30 K. The Δ*V* of κ-(ET)$_2$Cu[N(CN)$_2$]Cl at 30 K was then estimated to be 5.4 × 10^{-7} cm^3 mol^{-1}. It was considered that Δ*V* nearly saturates at 30 K, which is well below the critical endpoint, T_c = 40 K.

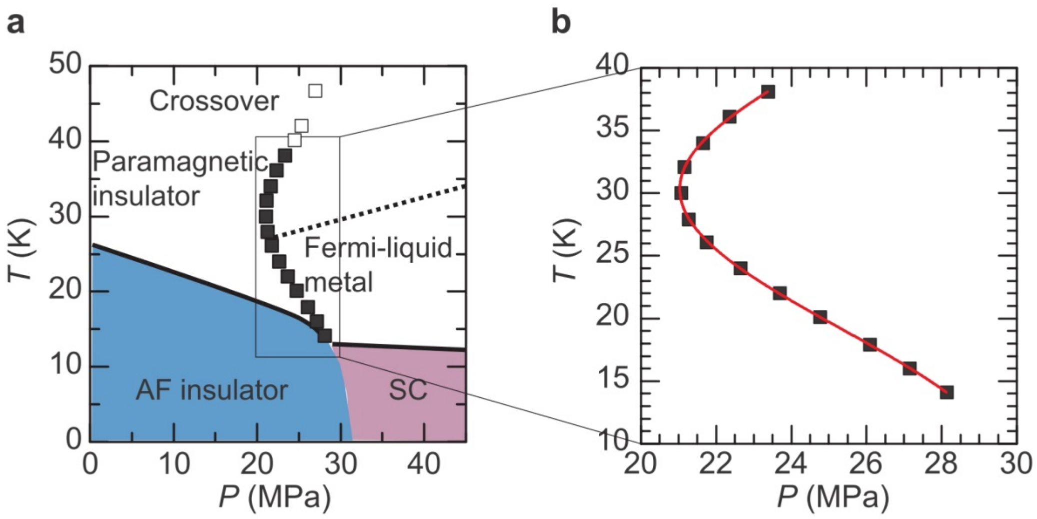

Supplementary Figure 5 | Pressure-temperature phase diagram of κ-$(ET)_2Cu[N(CN)_2]Cl$. a, Phase diagram near the Mott boundary. The closed and open squares indicate the pressures at the first-order Mott transition and the Mott crossover, respectively. The data points were reproduced from Supplementary Ref. 6. **b,** The first-order transition curve that fits the data points was reproduced from Supplementary Ref. 6.

Supplementary Note 4

Sheet resistance near the Mott boundary

As κ-$(ET)_2Cu_2(CN)_3$ is structured by the stacking of two-dimensional layers of d = 1.45 nm in thickness, the sheet resistance of the individual layer, R_{sheet}, at the resistance plateau is given by $R_{\mathrm{sheet}} = \rho_{\mathrm{plateau}} \times d^{-1} = R_{\mathrm{plateau}} \times \rho_{\mathrm{amb,RT}} / R_{\mathrm{amb,RT}} \times d^{-1}$, where ρ_{plateau} is the resistivity at the plateau, and $\rho_{\mathrm{amb,RT}}$ ($R_{\mathrm{amb,RT}}$) is the resistivity (resistance) at room temperature under ambient pressure. Using the values of R_{plateau} = 60 Ω, $R_{\mathrm{amb,RT}}$ = 14.6 Ω and $\rho_{\mathrm{amb,RT}}$ = 30 mΩcm (Supplementary Ref. 8), we obtain $R_{\mathrm{sheet}} = 8.2 \times 10^5$ Ω = 33 × h/e^2.

Supplementary Note 5

The Clausius-Clapeyron analysis taking into account the temperature dependence of the volume jump ΔV

In the main text, we assume that ΔV is temperature independent well below T_c because ΔV is reasonably expected to saturate at low temperatures. Here, we conduct a Clausius-Clapeyron analysis for κ-$(ET)_2Cu_2(CN)_3$ and κ-$(ET)_2Cu[N(CN)_2]Cl$, taking into account the temperature dependence of ΔV. There are no direct experimental results of $\Delta V(T)$. We then adopted the temperature profile of the order parameter of the two-dimensional Ising model as the temperature dependence of ΔV because the two-dimensional Mott transition can be mapped to the two-dimensional Ising model. Supplementary Figs. 6a and 6d show the expected temperature dependencies of ΔV for κ-$(ET)_2Cu_2(CN)_3$ and κ-$(ET)_2Cu[N(CN)_2]Cl$, respectively. It is seen that the ΔV varied only at the highest measured temperatures, resulting in no practical differences between Figs. 4a-d (assuming ΔV = const.) and Supplementary Figs. 6 b, c, e, and f (assuming the abovementioned T-dependence of ΔV), respectively.

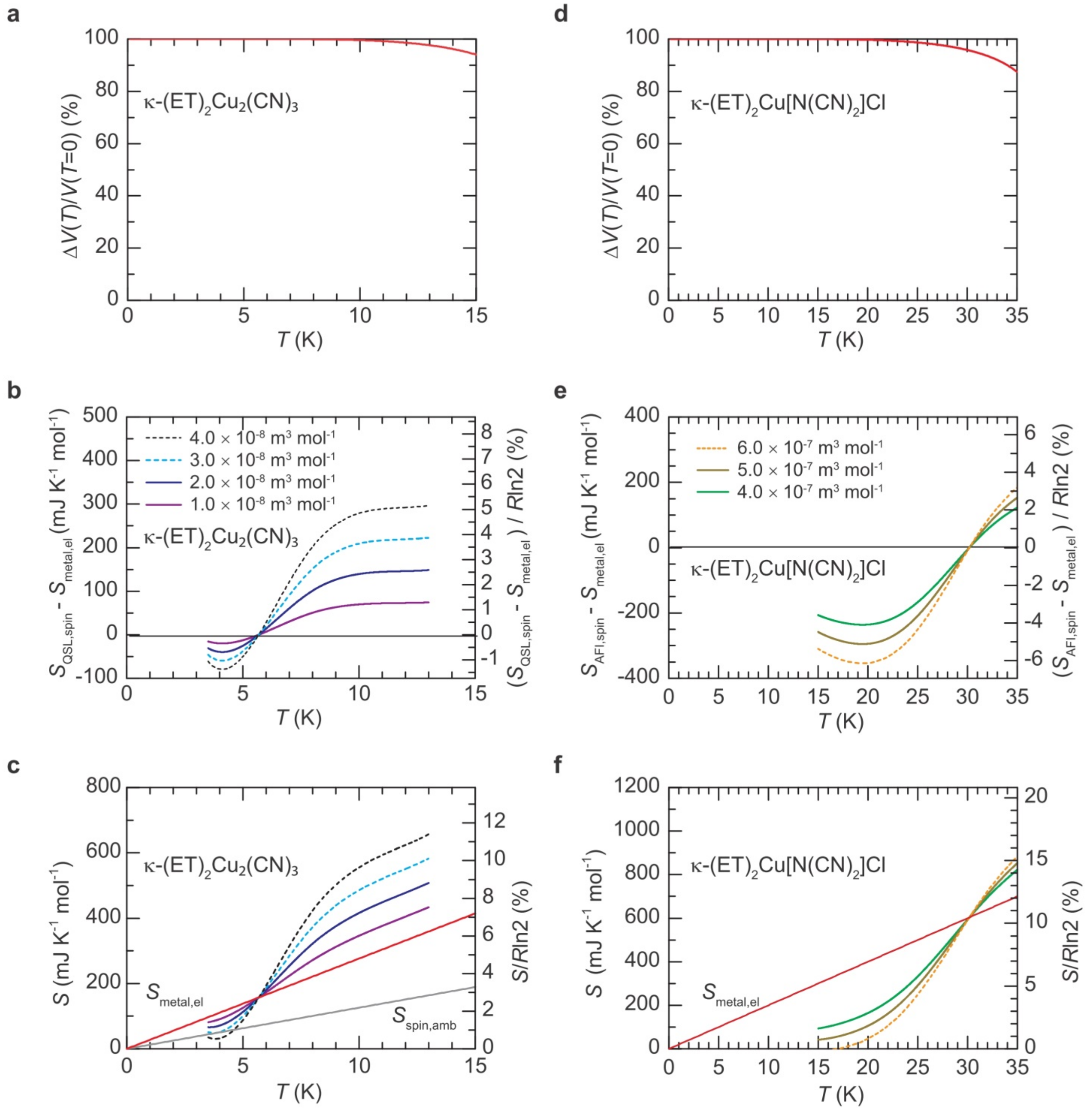

Supplementary Figure 6 | Thermodynamic properties of κ-$(ET)_2Cu_2(CN)_3$ and κ-$(ET)_2Cu[N(CN)_2]Cl$ on the Mott boundary. The temperature dependencies of $\Delta V(T)/\Delta V(T = 0\ \mathrm{K})$ for κ-$(ET)_2Cu_2(CN)_3$ and κ-$(ET)_2Cu[N(CN)_2]Cl$ were assumed to follow that of the order parameter of the two-dimensional Ising model, as shown in (**a**) and (**d**), respectively. The temperature dependencies of (**b**) $S_{\mathrm{QSL,spin}} - S_{\mathrm{metal,el}}$ and (**c**) $S_{\mathrm{QSL,spin}}$ for κ-$(ET)_2Cu_2(CN)_3$ and (**e**) $S_{\mathrm{AFI,spin}} - S_{\mathrm{metal,el}}$ and (**f**) $S_{\mathrm{AFI,spin}}$ for κ-$(ET)_2Cu[N(CN)_2]Cl$ are shown for several values of parameter $\Delta V(T = 0\ \mathrm{K})$. R is the universal gas constant. The dashed lines lead to unphysical entropy for $S < 0$ or $dS/dT < 0$ and provide upper bounds for the ΔV values. The grey line in (c) indicates $S_{\mathrm{spin,amb}} = \gamma_s T$, with $\gamma_s = 12.6$ mJ K^{-2} mol^{-1} (Supplementary Ref. 9). The red lines in (c) and (f) show $S_{\mathrm{metal,el}} = \gamma T$, where $\gamma = 27.6$ mJ K^{-2} mol^{-1} for κ-$(ET)_2Cu_2(CN)_3$, and $\gamma = 20$ mJ K^{-2} mol^{-1} for κ-$(ET)_2Cu[N(CN)_2]Cl$. The former γ value for κ-$(ET)_2Cu_2(CN)_3$ under pressure was deduced using the ^{13}C NMR Knight shift and hyperfine coupling constant (Supplementary Ref. 10). For the latter γ value, we refer to the value of the partially deuterated κ-$(ET)_2Cu[N(CN)_2]Br$ (Supplementary Ref. 11), which is a Mott insulator on the verge of the Mott transition. We also assumed that $T_c = 18$ K for κ-$(ET)_2Cu_2(CN)_3$ and $T_c = 38$ K for κ-$(ET)_2Cu[N(CN)_2]Cl$.

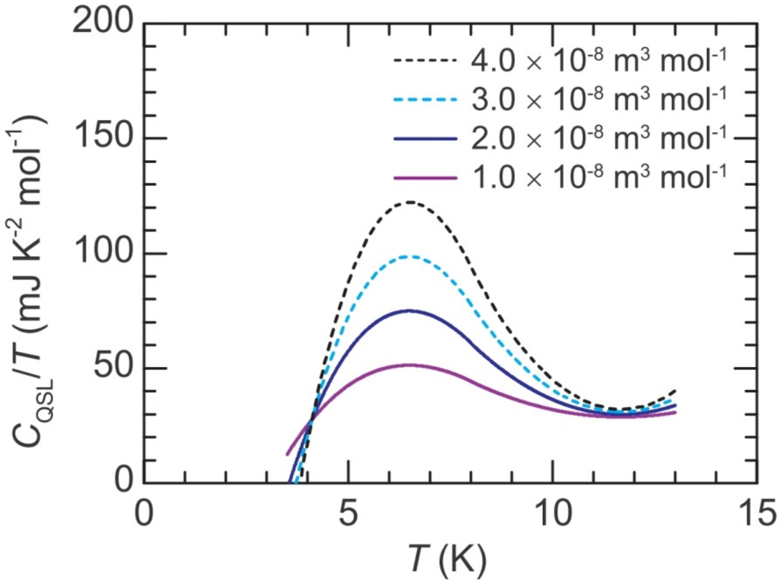

Supplementary Figure 7 | Temperature dependence of the specific heat divided by temperature of the spin liquid on the Mott boundary of κ-(ET)$_2$Cu$_2$(CN)$_3$. The specific heat divided by temperature of the spin liquid on the Mott boundary C_{QSL}/T was deduced through the relation $C_{QSL}/T = \partial S_{QSL,spin}/\partial T = \gamma + \Delta V \times (\partial^2 P_{jump}/\partial T_{jump}^2)$, with ΔV as a parameter.

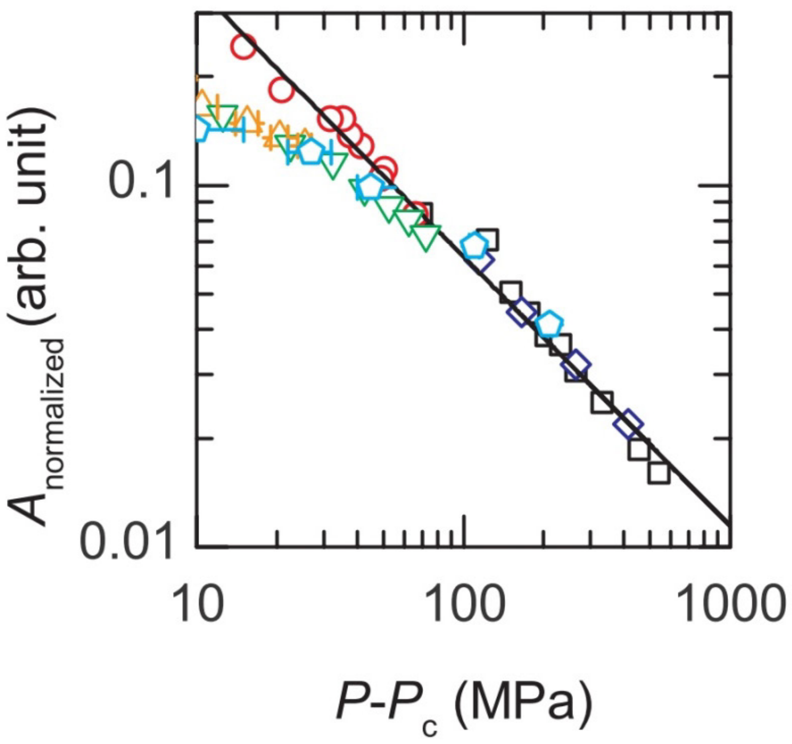

Supplementary Figure 8 | Pressure dependencies of the T^2 coefficient of resistance near a critical pressure for various materials. The T^2 coefficients of resistance A for κ-$(ET)_2Cu_2(CN)_3$ (red circles, black squares, and blue diamonds, P_c = 135 MPa), κ-$(ET)_2Cu[N(CN)_2]Cl$ (green inverted triangles, P_c = 25 MPa, from Supplementary Ref. 12), κ-$(ET)_2Cu[N(CN)_2]Br$ (cyan pentagons, P_c = -10 MPa ± 5 MPa (Supplementary Ref. 13), from Supplementary Ref. 14), and deuterated κ-$(ET)_2Cu[N(CN)_2]Br$ (orange triangles, P_c = 4.5 ± 1.5 MPa) are shown. For comparison, the As for different materials were normalized to each other at high pressures.

Supplementary Note 6

Estimation of charge gap

Because the charge gap Δ can be extracted from the relation $R \propto \exp(\Delta/2T)$ at $2T \ll \Delta$, $2d(\ln R)/d(1/T)$ directly yields the value of the charge gap. We plot the $2d(\ln R)/d(1/T)$ as a function of $1/T$ in Supplementary Fig. 9. The charge gap is defined by the dashed lines shown in Supplementary Fig. 9. The error of the fitting constant is indicated in Fig. 1c and Fig. 3 in the main text. At $P > 118$ MPa, it is difficult to extract the value of charge gap from the $2d(\ln R)/d(1/T)$ because $2d(\ln R)/d(1/T)$ is much less than $2T$. However, it is obvious that the temperature dependence of resistance at $P > 118$ MPa showed vanishingly small gaps that were much smaller than the lowest measured temperature of ~ 2 K.

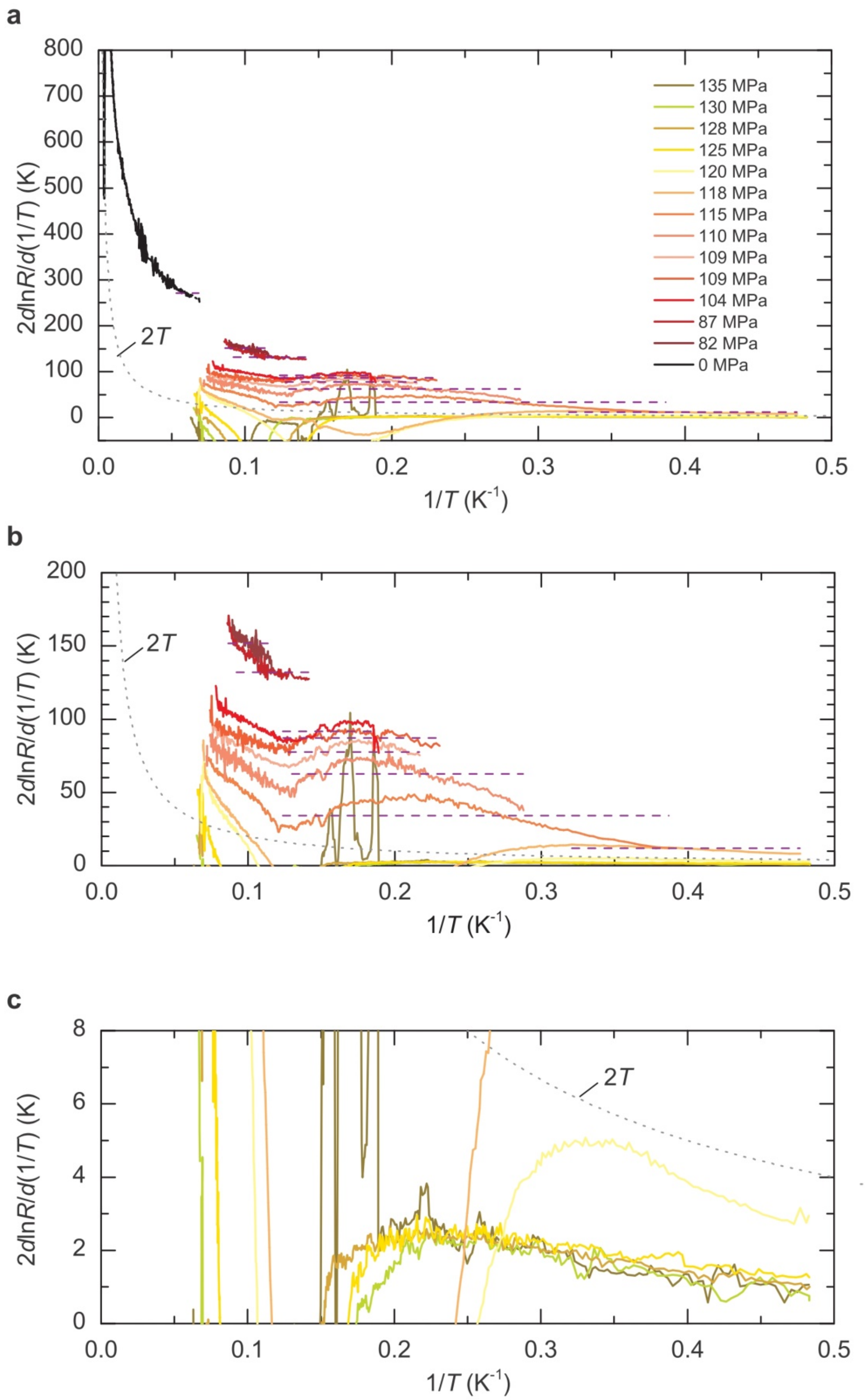

Supplementary Figure 9 | $2d(\ln R)/d(1/T)$ vs. $1/T$ for various pressures. $2d(\ln R)/d(1/T)$ is plotted as a function of $1/T$ for various pressures with different magnifications **(a, b, and c)**. The grey dotted curves indicate $2T$, and the purple dashed lines indicate the fit values for the activation energy.

Supplementry References

1. Driessen, A., van der Poll, E. & Silvera, I. F. Equation of state of solid ^{4}He. *Phys. Rev. B* **33,** 3269–3288 (1986).
2. Andraka, B. *et al.* Specific heat in high magnetic field of κ-di[bis(ethylenedithio)tetrathiafulvalene]-di(thiocyano)cuprate [κ-$(ET)_2Cu(NCS)_2$]: Evidence for strong-coupling superconductivity. *Phys. Rev. B* **40,** R11345–R11347 (1989).
3. Kanoda, K. Metal–insulator transition in κ-$(ET)_2X$ and $(DCNQI)_2M$: two contrasting manifestation of electron correlation. *J. Phys. Soc. Japan* **75,** 051007 (2006).
4. Andraka, B. *et al.* Specific heat of the high T_c organic superconductor κ-$(ET)_2Cu[N(CN)_2]Br$. *Solid State Commun.* **79,** 57–59 (1991).
5. Uehara, T., Ito, M., Taniguchi, H. & Satoh, K. Superconducting fluctuation of the layered organic superconductor κ-$(BEDT\text{-}TTF)_2Cu[N(CN)_2]Br$ in magnetic susceptibility. *J. Phys. Soc. Japan* **82,** 073706 (2013).
6. Kagawa, F., Itou, T., Miyagawa, K. & Kanoda, K. Transport criticality of the first-order Mott transition in the quasi-two-dimensional organic conductor κ-$(BEDT\text{-}TTF)_2Cu[N(CN)_2]Cl$. *Phys. Rev. B* **69,** 064511 (2004).
7. Gati, E. *et al.* Breakdown of Hooke's law of elasticity at the Mott critical endpoint in an organic conductor. *Sci. Adv.* **2,** e1601646 (2016).
8. Furukawa, T., Miyagawa, K., Taniguchi, H., Kato, R. & Kanoda, K. Quantum criticality of Mott transition in organic materials. *Nat. Phys.* **11,** 221–224 (2015).
9. Yamashita, S. *et al.* Thermodynamic properties of a spin-1/2 spin-liquid state in a κ-type organic salt. *Nat. Phys.* **4,** 459–462 (2008).
10. Shimizu, Y. *et al.* Pressure-induced superconductivity and Mott transition in spin-liquid κ-$(ET)_2Cu_2(CN)_3$ probed by ^{13}C NMR. *Phys. Rev. B* **81,** 224508 (2010).
11. Nakazawa, Y., Taniguchi, H., Kawamoto, A. & Kanoda, K. Electronic specific heat at the boundary region of the metal-insulator transition in the two-dimensional electronic system of κ-$(BEDT\text{-}TTF)_2Cu[N(CN)_2]Br$. *Phys. Rev. B* **61,** R16295–R16298 (2000).
12. Limelette, P. *et al.* Mott Transition and Transport Crossovers in the Organic Compound κ-$(BEDT\text{-}TTF)_2Cu[N(CN)_2]Cl$. *Phys. Rev. Lett.* **91,** 016401 (2003).
13. Sasaki, T., Yoneyama, N. & Kobayashi, N. Mott transition and superconductivity in the strongly correlated organic superconductor κ-$(BEDT\text{-}TTF)_2Cu[N(CN)_2]Br$. *Phys. Rev. B* **77,** 054505 (2008).
14. Strack, C. *et al.* Resistivity studies under hydrostatic pressure on a low-resistance variant of the quasi-two-dimensional organic superconductor κ-$(BEDT\text{-}TTF)_2Cu[N(CN)_2]Br$: Search for intrinsic scattering contributions. *Phys. Rev. B* **72,** 054511 (2005).
